# First report of *Nocardia wallacei* infection in an immunocompetent patient in Zhejiang province

**DOI:** 10.1515/biol-2022-0891

**Published:** 2024-06-18

**Authors:** Wei Pan, Bingqian Zhuo, Sumei Wang, Jieping Long, Wei Xu, Mengyuan Chen, Xin Hong, Yumei Ge

**Affiliations:** Department of Clinical Laboratory, Haiyan People’s Hospital, Haiyan, Zhejiang, 314300, China; School of Public Health, Hangzhou Medical College, Hangzhou, 310053, China; Laboratory Medicine Center, Department of Clinical Laboratory, Zhejiang Provincial People’s Hospital (Affiliated People’s Hospital), Hangzhou Medical College, Hangzhou, Zhejiang, 310014, China; School of Medical Technology and Information Engineering, Zhejiang Chinese Medical University, Hangzhou, Zhejiang, 310053, China; Department of Green Pharmaceutical Collaborative Innovation Center, School of Pharmacy, Zhejiang University of Technology, Hangzhou, Zhejiang, 310014, China; The Second Clinical Medical College, Zhejiang Chinese Medical University, Hangzhou, Zhejiang, 310053, China

**Keywords:** nocardiosis, *Nocardia wallac*ei, linezolid, immunocompetent, MALDI-TOF MS

## Abstract

Nocardiosis is an infectious disease caused by *Nocardia* spp., mainly affecting immunocompromised hosts. *Nocardia* infection is not common; especially *Nocardia wallacei* infection is even rarer. The patient, female, 61 years old, farmer, has been working in the field for a long time and has normal immune function. Her main clinical manifestation was persistent back pain. Chest-enhanced computed tomography showed pulmonary inflammation. Rare pathogen *Nocardia wallac*ei was detected in alveolar lavage fluid using matrix-assisted laser destructive ionization time-of-flight mass spectrometry. She received treatment with linezolid and was discharged after her condition improved.

## 1 Introduction

In recent years, with the increase of HIV infection, solid organ transplantation, hematopoietic stem cell transplantation, autoimmune disease patients treated with corticosteroids, hematological malignancies, and lung structural and functional damage, Nocardiosis has begun to appear frequently in the public health [1]. Nocardiosis is a rare bacterial infection caused by *Nocardia* that usually affects individuals with low immunity and has a high incidence rate and mortality [2,3]. Nocardiosis mainly manifests as fever, cough, sputum, chest pain, and other symptoms, which has no characteristic clinical manifestations compared with other infections and make it challenging to diagnose in clinical practice [4]. *Nocardia* is a group of aerobic actinomycetes, mostly saprophytic and mainly distributed in soil. It is an opportunistic pathogenic bacterium that primarily invades the body through the respiratory tract or wounds, causing purulent infections. In severe cases, it can spread to multiple organs through the bloodstream [2,4,5]. Currently, over 90 species of *Nocardia* have been described, of which approximately 30 can cause human diseases [4,6]. *Nocardia cyriacigeorgica* and *Nocardia farcinica* are considered the main pathogenic species worldwide, while in China, *Nocardia asteroides* is commonly found [7]. *Nocardia wallacei* was initially characterized in 2008 through molecular phylogenetic analysis of the 16S ribosomal RNA (rRNA) gene, the 65-kilodalton heat shock protein (hsp65) gene, and the secA1 gene, substantiated by DNA-DNA hybridization techniques. [8]. To date, a total of nine documented clinical cases of infections caused by *Nocardia wallacei* have been globally reported [8,9,10,11,12,13,14,15,16]. It has become one of the emerging species of human pathogenic *Nocardia* and has brought therapeutic challenges, which have attracted researchers’ attention due to its high levels of antibiotic resistance, especially to the aminoglycosides. There are fewer types of antibiotics available for the treatment of *Nocardia wallacei* compared to other *Nocardia* spp. of antibiotic therapy [17]. Trimethoprim-sulfamethoxazole alone or in combination is still the first line and preferred empirical medication for the treatment of all types of nocardiosis, including *Nocardia wallacei* infections, due to the high sensitivity of the *Nocardia* genus to sulfonamide [2,18]. We report the first patient with *Nocardia wallacei* infection who was allergic to sulfamethoxazole and improved after treatment with linezolid in Zhejiang.

## 2 Case presentation

### 2.1 Clinical features

**Figure 1:**
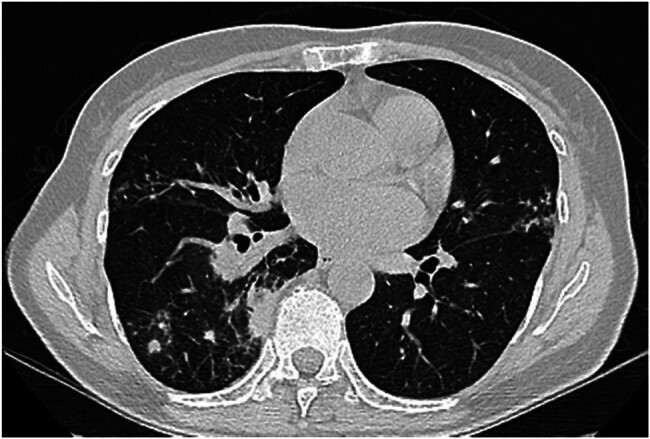
CT image of the patient’s lungs.


A 61-year-old female farmer was admitted due to persistent back pain. Abnormal elevation of neutrophils (79.2%) and C-reactive protein (46.5 mg/L) were indicated by blood routine examination (Table 1). Computed tomography (CT) scan showed pulmonary inflammation with bronchiectasis (Figure 1).

**Table 1:** Clinical and laboratory indicators of the patient

Laboratory/clinical indicators	Measurements	Normal interval
White blood cells	9.2 × 10^9^/L	3.5–9.5 × 10^9^/L
Lymphocytes	14.10%	20–50%
Monocytes	6.00%	3–10%
Neutrophils	79.20%	40–75%
Eosinophils	0.4 × 10^9^/L	0.4–8.0 × 10^9^/L
Basophils	0.3 × 10^9^/L	0.0–1.0 × 10^9^/L
Red blood cells	3.79 × 10^12^/L	3.8–5.1 × 10^12^/L
Hemoglobin	120 g/L	115–150 g/L
Platelets	204 × 10^9^/L	125–350 × 10^9^/L
C-reactive protein	46.5 mg/L	0.0–8.0 mg/L
Blood pressure	132/72 mmHg	
Respiratory rate	20 Breaths/min	
Heart rate	91 Beats/min	
Temperature	37.0°C	

**Informed consent:** Informed consent has been obtained from all individuals included in this study.
**Ethical approval:** The research related to human use has complied with all the relevant national regulations, and institutional policies and in accordance with the tenets of the Helsinki Declaration, and has been approved by the Ethics Committee of Haiyan People’s Hospital. (Ethics Committee Approval of Biomedical Research Involving Humans, Approval No.: 202310).

## 3 Etiological examination

**Figure 2:**
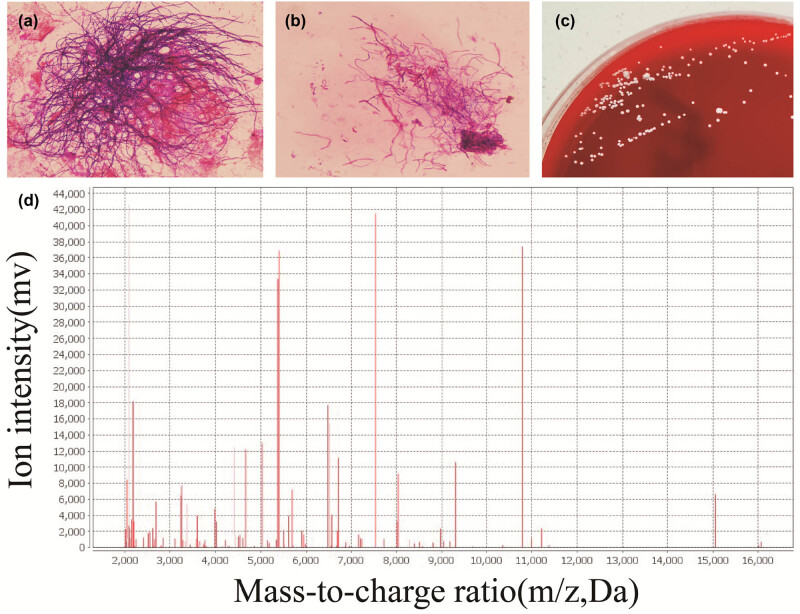
Etiological examination: (a) Gram-positive bacteria were observed by Gram staining under an oil mirror (×1,000 magnification); (b) acid-fast staining under oil mirror (×1,000 magnification); (c) wrinkled, dry, and white colonies of different sizes were observed by culture on the blood agar plate; (d) MALDI-TOF MS confirmed it was *Nocardia wallacei*.

Microscopic characteristics of the patient’s alveolar lavage fluid were determined by Gram staining. A large number of Gram-positive filamentous bacteria were observed to be arranged radially, with varying shades of staining resembling beads under oil microscopy, indicating that it may be of the genus Nocardia (Figure 2a). Acid-fast staining was weakly positive under an oil microscope (Figure 2b). White colonies of varying sizes, dry or waxy colonies, with wrinkles and particles on the surface were observed after 3 days of aerobic incubation on a blood agar plate (containing blood components that provide ample nutrients and growth factors, suitable for the growth of most aerobic and facultative anaerobic bacteria) of alveolar lavage fluid (Figure 2c). The *Nocardia wallacei* colony was picked out with sterile toothpicks or inoculation rings, and the bacteria were evenly smeared on the target plate dedicated to Matrix-assisted laser desorption ionization-time of flight mass spectrometry (MALDI-TOF MS) to form a thin layer to avoid overlapping. Formic acid was added dropwise, and the plate was dried. Matrix liquid was then added dropwise. After the target plate was dried, it was detected on the machine. In the mass spectrometer, the sample was separated by effective ionization to form a clear fingerprint of bacteria specificity. MALDI-TOF MS was used for colony identification and it indicated *Nocardia wallacei* with a 99.9% confidence (Figure 2d).

## 4 Treatment and prognosis

Due to the patient’s complaint about previous allergies to trimethoprim-sulfamethoxazole, we used an intravenous infusion of 0.6 g of linezolid every 12 h for anti-infection treatment of *Nocardia wallacei*. After 18 days, the patient’s inflammatory indicators returned to normal and the condition improved significantly. The intravenous injection of linezolid was stopped and the patient was discharged. She was recommended to continue taking oral linezolid for treatment and regularly return to the respiratory clinic for follow-up. The patient’s current prognosis is excellent according to our latest follow-up (6 months since the first treatment of linezolid).

## 5 Discussion

The clinical manifestations and imaging features of nocardiosis are nonspecific, so it may be misdiagnosed as other infections including lung abscess, tuberculosis, bacterial pneumonia, and pulmonary aspergillosis [19]. Pulmonary infection is the most common manifestation of nocardiosis [20]. The lung CT of our case showed inflammation, and the lung infection was likely acquired by inhaling *Nocardia* from dust or soil. The patient is a farmer who has been working in the field for a long time, so she may have the potential risk of infection by pathogens in the soil. Although *Nocardia* has been reported in medical literature, *Nocardia wallacei* infection in humans is rare and easily misdiagnosed. A total of nine cases of *Nocardia wallacei* infection were reported in the world, including four cases of immunocompetent patients and five cases of immunocompromised patients (one case of diabetes mellitus, two cases of HIV infection, one case of lymphoma, and one case of oral carcinoma; Table 2). *Nocardia* is identified as a bacterium that is aerobic, gram-positive, partially acid-fast, resistant to lysozyme, and positive for catalase [8]. It displays a unique cellular morphology with beaded, branching characteristics. Diagnosis and identification of *Nocardia* are crucial due to the requirement for specific antibiotics in treatment, with antibiotic susceptibility varying among strains. Initially, careful examination of pus, sputum, or other exudates and secretions for pigment granules is conducted. Smears or crush preparations of these samples undergo Gram staining and acid-fast staining. The presence of Gram-positive, branching filaments that are partially acid-fast may suggest *Nocardia*, albeit requiring differentiation from Mycobacterium tuberculosis. In this case, upon bronchoalveolar lavage (BAL) fluid smear microscopy, numerous Gram-positive, filamentous bacteria arranged radially were observed, appearing like beads under oil immersion microscopy with variations in staining intensity, indicative of possible *Nocardia* species. A weakly positive result on modified acid-fast staining further supported this preliminary identification. Culturing of the BAL fluid yielded colonies of varied sizes, with a wrinkled, dry, and white appearance. In recent years, the number of microbial species that MALDI-TOF MS can routinely identify has increased, and now, it can reliably identify closely related species, including Nocardia [21]. MALDI-TOF MS has become the reference method for routine identification of bacterial isolates in clinical microbiology laboratories worldwide [22]. MALDI-TOF MS analysis identified the isolate as *Nocardia wallacei*, thereby consolidating the diagnosis of a *Nocardia* infection. A German pathologist discovered in 1932 that berberine had an inhibitory effect on Streptococcus, marking the earliest discovery of the antimicrobial effect of sulfonamide drugs [15]. Sulfonamide drugs have been in clinical use for nearly 50 years, exhibiting advantages such as a broad spectrum of antibacterial activity, stability, low cost, high production yield, and convenient usage [23]. The combination of trimethorprim-sulfamethoxazole remains one of the preferred antibiotic treatments for *Nocardia* infections [24,25]. In this case, the patient exhibited an allergic reaction to sulfonamide drugs, rendering them unsuitable for treatment. Consequently, we opted for linezolid to improve the patient’s condition. Linezolid is a synthetic antimicrobial agent, belonging to the oxazolidinone class of antibiotics. It finds clinical utility in treating infections caused by Gram-positive aerobic bacteria including *Nocardia wallacei* [14]. Masahiro Toyokawa et al. found in their study of 146 strains of *Nocardia* receiving AST that linezolid was the most active drug among all species and did not exhibit *in vitro* resistance. In the absence of species identification, linezolid is a preferential empirical treatment option [16]. This is the first case report of *Nocardia wallacei* infection in Zhejiang, China, and it confirmed that it can cause pulmonary infection in immunocompetent patients.

**Table 2:** Reported cases of *N. wallacei* in immunocompetent and immunocompromised patients

Year	Country	Age	Gender	① Infection types	Associated risk factors/underlying disease	Symptoms and radiological findings	Targeted therapy	Outcome	Reference
				② Sample sources					
				③ Identification method					
2023	China	61	Female	Lungs	Immunocompetent	Persistent back pain, CT: pulmonary inflammation with bronchiectasis	Linezolid, 0.6 g, 12 h	Rehabilitation	Present case
				BALF					
				MALDI-TOF MS					
2023	India	75	Male	Lungs	Type 2 diabetes mellitus and epilepsy	Acute febrile, lower respiratory tract infection, bilateral pneumonia, severe hypoxia, fever and shortness of breath cough and weakness. X-ray: bilateral nodular opacities and infiltrates, along with areas of consolidation. CT: multiple heterogeneously enhancing lesions of bilateral lung fields with consolidation of the right lower lobe.	None	Died	Ranjan et al. [9]
				Sputum					
				16s rRNA sequencing					
2023	China	59	Female	Lungs and subcutaneous	Immunocompetent	Paroxysmal cough, mucus sputum, pain in the chest and back, lump on back has grown larger. CT: a right upper lung nodule, destruction of the right lung, the bone destruction of the right ribs	Imipenem 1.0 g ivgtt, Clarithromycin 0.5 g po, Doxycycline 0.1 g po, 12 h to suppress *Mycobacterium abscessus* TMP-SMX 0.96 g po, 6 h to suppress *N. wallacei.*	Rehabilitation	Qin et al. [10]
				Skin pus					
				mNGS					
2022	Italy	80	Male	Brain and lungs	Lymphoplasmacytic lymphoma	Progressive ideomotor impairment, disorientation, and memory deficiency. CT scan and brain MRI showed necrotic lesions with associated oedema in the left frontal and parietal lobe. CT: multiple bilateral nodules and consolidations, along with bilateral pleural effusion.	TMP/SMX and linezolid (600 mg, 12 h).	Rehabilitation	Palomba et al. [11]
				Brain specimen					
				Not mentioned					
2020	American	46	Male	Lungs and brain	Immunocompetent	Worsening visual acuity, diplopia, left arm numbness, left facial droop and left hemiparesis. CT: abdomen and pelvis showed a 5 × 5 cm thick walled left upper lobe lung cavitary lesion and a cystic adrenal mass with necrotic supraclavicular and mediastinal lymphadenopathy	TMP/SMX and aminoglycosides (Amikacin, Tobramycin)	Not mentioned	Sithamraju et al. [12]
				Purulent fluid					
				16s rRNA sequencing					
2018	Mexico	18	Female	Skin and soft tissue	Immunocompetent	Increased volume and draining nod-ules of the left leg, tinea versicolor, extensive seborrheic dermatitis, and disseminated molluscum contagiosum, fever and low back pain. MRI: a collection in the left psoas	Linezolid 600 mg, 3 months	Rehabilitation	Welsh et al. [13]
				Not mentioned					
				Not mentioned					
2016	Mexico	43	Female	Lungs	HIV	Presenting a breathing failure. Not mentioned	Not mentioned	Not mentioned	González-Nava et al. [14]
				Sputum					
				16s rRNA sequencing					
2013	France	62	Female	Lung and brain	Immunocompetent	Headache, breathing failure and right pleuritic CT: disseminated pulmonary nodulesa. MRI: brain abscess	Linezolid 600 mg/12 h; ciprofloxacin, 500 mg/8 h, 2weeks; TMP-SMX and linezolid/12 h, 4weeks	Rehabilitation	Cassir et al. [15]
				BALF					
				16s rRNA sequencing					
2013	Arabia	54	Male	Lung	HIV	Chronic pulmonary illness, CT: multiple nodular lesions	Not mentioned	Rehabilitation	Hamid et al. [16]
				BALF					
				16s rRNA sequencing					
2008	American	57	Male	Pleura and brain	Oral carcinoma, pneumocystis jirovecii pneumonia	With a 3-week history of chills, fever, cough, and left pleuritic pain.	Trimethoprim-sulfamethoxazole, amikacin and so on	Died	Conville et al. [8]
				Pleural fluid and sputum					
				16s rRNA sequencing					

## 6 Conclusion

We report the first case of pulmonary infection caused by *Nocardia wallacei* in a patient with normal immune function in Zhejiang province. This *Nocardia wallacei* isolate was successfully identified by MALDI-TOF MS, which provided a new option for the identification of *Nocardia*. After treatment with linezolid, inflammation indicators of the patient returned to normal and improved. Including this case, only 10 cases of *Nocardia wallacei* infection have been reported so far, thus more cases are needed for the investigation of spatiotemporal distribution and drug sensitivity of *Nocardia wallacei* isolates as well as clinical manifestation, morbidity and mortality of the patients.
